# The Network Structure of Human Personality According to the NEO-PI-R: Matching Network Community Structure to Factor Structure

**DOI:** 10.1371/journal.pone.0051558

**Published:** 2012-12-20

**Authors:** Rutger Goekoop, Jaap G. Goekoop, H. Steven Scholte

**Affiliations:** 1 Department of Mood Disorders, PsyQ Psychomedical Programs, The Hague, The Netherlands; 2 Department of Psychiatry, Leiden University Medical Center, Leiden University, Leiden, The Netherlands; 3 Cognitive Neuroscience Group, Department of Psychology, University of Amsterdam, Amsterdam, The Netherlands; 4 Parnassia Bavo Goup, The Hague, The Netherlands; 5 Brain and Cognition Priority Program, University of Amsterdam, Amsterdam, The Netherlands; Uni. of South Florida, United States of America

## Abstract

**Introduction:**

Human personality is described preferentially in terms of factors (dimensions) found using factor analysis. An alternative and highly related method is network analysis, which may have several advantages over factor analytic methods.

**Aim:**

To directly compare the ability of network community detection (NCD) and principal component factor analysis (PCA) to examine modularity in multidimensional datasets such as the neuroticism-extraversion-openness personality inventory revised (NEO-PI-R).

**Methods:**

434 healthy subjects were tested on the NEO-PI-R. PCA was performed to extract factor structures (FS) of the current dataset using both item scores and facet scores. Correlational network graphs were constructed from univariate correlation matrices of interactions between both items and facets. These networks were pruned in a link-by-link fashion while calculating the network community structure (NCS) of each resulting network using the Wakita Tsurumi clustering algorithm. NCSs were matched against FS and networks of best matches were kept for further analysis.

**Results:**

At facet level, NCS showed a best match (96.2%) with a ‘confirmatory’ 5-FS. At item level, NCS showed a best match (80%) with the standard 5-FS and involved a total of 6 network clusters. Lesser matches were found with ‘confirmatory’ 5-FS and ‘exploratory’ 6-FS of the current dataset. Network analysis did not identify facets as a separate level of organization in between items and clusters. A small-world network structure was found in both item- and facet level networks.

**Conclusion:**

We present the first optimized network graph of personality traits according to the NEO-PI-R: a ‘Personality Web’. Such a web may represent the possible routes that subjects can take during personality development. NCD outperforms PCA by producing plausible modularity at item level in non-standard datasets, and can identify the key roles of individual items and clusters in the network.

## Introduction

Currently, the most influential way of looking at human personality is the multidimensional trait approach [1]. In this view, the term ‘personality’ refers to a set of perceptions, inner experiences and behavioral traits that a person may possess, which are temporally stable and show little variance in time. A particular trait (such as curiosity) is held commonly amongst large numbers of subjects within a population, but the extent to which that trait is present necessarily differs between subjects. Personality has been described in several ways, which broadly include categorical and multidimensional descriptions [2]. Multidimensional descriptions are favored by researchers and increasingly by clinicians, because of their validity, reliability, and descriptive power [3], [4]. In multidimensional descriptions of personality, large numbers of specific personality characteristics (e.g. curiosity, impulsivity, a need for speeding, easy fatigability, irritability, and worrying over trifles) are grouped together based on their tendency to co-occur (covary). Thus, a smaller set of global variables is identified (e.g. ‘Openness to experience’, ‘Neuroticism’) that explains most of the variance within the larger set of measured variables. These global variables are called ‘factors’ or ‘dimensions’, and the entire set of factors that is found in a dataset is called its ‘factor structure’. Factor structures are typically identified using factor analysis [5]. In many cases, factor analysis involves principal component analysis (PCA), which aims to find a set of orthogonal (i.e. uncorrelated) factors that are called ‘principal components’. These represent the main independent sources of variance in the dataset. Principal components can be added linearly to reconstruct the variance that is contained in the dataset at large.

In personality research, PCA is the preferred type of factor analysis. A single component score (or factor score) can be calculated for each principal component by averaging the scores on the covarying subvariables that define the component. Thus, a limited set of factor scores (a factor profile) can be used to provide a compact description of individual subjects, hence its attraction in personality research. Factor profiles can be used to define the personality of individual subjects or groups. Such profiles allow for predictions of specific human behaviors (e.g. cigarette smoking, obesity, divorce) under certain circumstances (e.g. stress) [6], [7]. Additionally, factor profiles can successfully predict measures of subjective wellbeing [8], disease prevalence and outcome [9], [10], accident-proneness, risk of injuries [11], and premature death [12].

Currently, the most influential multidimensional descriptive model of human personality is the five-factor model [13], [14], of which the Neuroticism-Extraversion-Openness Personality Inventory Revised (NEO-PI-R) is the most commonly used implementation [15]. The NEO-PI-R is a self-rating scale that consists of 240 items representing personality traits that are scored on a six-point Likert scale. PCA has been used to examine the factor structure of a standardized (norm) dataset of healthy American subjects. This has identified five basic factors for personality that explain an optimal amount of variance in the original data: Neuroticism, Extraversion, Openness, Agreeableness and Conscientiousness. These factors are consistently found in different populations and countries worldwide, although some differences exist between the various countries and cultures [16]. For each factor of the NEO-PI-R, six facets have been defined that measure different subtraits of the larger factor (e.g. n1–n6 represent Anxiety, Anger, Depression, Self-Consciousness, Immoderation and Vulnerability). Facets represent an intermediate level of organization in between items and factors. Although the exact nature and number of facets does not follow immediately from empirical measures, there is considerable empirical support to assume an intermediate level of organization in between items and factors [17]. Facets have been extensively tested for their reliability and predictive power [18]. The use of facets increases the resolution of personality measurements and seem to add to the accuracy of predictions of human behavior [10]. Overall, the NEO-PI-R is amongst the best supported personality scales used worldwide and will be used as a basis for clinical diagnosis of personality disorders in the next edition of the diagnostic and statistical manual (DSM-5), the main book of reference for clinical diagnosis of psychiatric disorders (http://www.DSM5.org).

Although the NEO-PI-R has an impressive record of empirical research behind it, its factors are based on PCA, which has several limitations. Most importantly, the mutual relationships between the items that make up the factors are not explicitly modeled, and hence disregarded. This may be unfortunate, since some of these interactions may have disproportionate importance when compared to others (e.g. some items may be correlated to many or fewer other items, show stronger or weaker correlations, explain more variance in factor scores, or have causal dominance over others). As a result, items, facets and factors of the NEO-PI-R are given the same weighting and diagnostic value. This may not be desirable, given the possibility that certain personality traits (such as neuroticism or agreeableness) have a disproportional importance in mediating healthy personality development. Additionally, PCA is known to produce erroneous results when performed at item level in smaller-than-standard personality datasets. This is because PCA requires large numbers of subjects (typically 6 times the number of items) to produce reliable factor structures [5]. Studies that aim to examine personality at item level therefore tend to be large and costly. Some workarounds exist for this problem, e.g. factor analysis in smaller datasets can be performed at facet-scores, which may produce reliable factor structures. Also, factor scores can be calculated from item scores using the international factor structure as a key. Despite their wide usage, however, such techniques inevitably lead to some loss of information or inaccuracy.

To address these issues, we examined whether a novel method to identify modularity in datasets (Network Community Detection - NCD) could compensate for some of the limitations of PCA. Interactions between variables in a dataset (such as correlations between item scores of the NEO-PI-R) can be viewed as a network of nodes (items) that communicate with each other through links (e.g. significant correlations). NCD involves the identification of dense cliques of interacting nodes within network graphs. In a recent study, it has been shown that network clusters produced by some NCD algorithms are both practically and theoretically very similar to those identified using PCA [19], [20]. Like PCA, NCD allows for the extraction of sets of covarying items or traits. PCA and NCD make use of the same covariance matrices to identify modularity in datasets, and the results are highly similar. Similar to higher-order factor analyses, NCD can examine the clustering of network clusters and identify super-clusters by examining the covariance between cluster scores. A fundamental difference between PCA and NCD, however, is that PCA identifies modules through an algebraic analysis of the variance, whereas NCD performs a geometrical analysis. Unlike factor analysis, network analysis provides a detailed view of the interactions that exist between individual items, facets or clusters [21]. Additionally, network analysis can attribute importance to individual network nodes and clusters that is based not only on their size or the amount of variance they explain in the dataset as a whole, but also on their strategic position within the network (e.g. nodes with high degrees (hubs) or betweenness centralities). NCD allows for the quantification of such central roles and examine the importance of individual items and clusters in directing the flow of information through the personality network, e.g. during personality development.

To examine the results of NCD when compared to PCA, we performed both types of analyses on data of 434 healthy subjects that completed the NEO-PI-R. Network graphs were created from correlation matrices representing the interactions between item scores and facet scores of the NEO-PI-R, in which nodes represent items or facets, links between the nodes represent significant correlations, and weights along the links are correlation coefficients (r). Given the theoretical similarity between PCA and NCD, we hypothesized that the FS of the NEO-PI-R dataset would closely match its NCS. NCS and FSs were first compared at ‘facet level’, i.e. between FSs and NCSs based on facet scores. Since PCA performed at facet level produces results that are comparable between standard and non-standard datasets such as the present dataset, NCS were expected to resemble both the FS of the current dataset and the norm structure. Next, we examined NCSs in networks representing correlations between item-scores. This was done to examine whether NCD, as opposed to PCA, would produce plausible modularity at item level in the current (non-standard) dataset. If NCD, in contrast to PCA, would find meaningful modules, this would indicate that NCD can out-perform factor analysis at this level. In case plausible modules would occur (i.e. resembling the results of PCA at item level in standard datasets), we expected the emergence of large-scale network clusters without an intermediate facet level, as is the case when using PCA at item level in standard datasets. Finally, we expected to obtain a richer view of the singular nature of individual items, facets and network clusters than provided by PCA.

To allow for a direct comparison between NCD and PCA, a matching procedure was used in which the match of NCS to FS was optimized. In order to find the best match, the global threshold of the personality network (i.e. the threshold for the significance of a link) was raised incrementally in the order of increasing levels of significance of links (‘incremental pruning’) until its NCS showed an optimal match with FS. Three different FSs were used as templates for matching: the FS of the standard (norm) dataset that contains 5 principal components (the ‘standard 5-FS’), as well as the FSs derived from the present dataset using both a 5-factor PCA (the ‘confirmatory 5-FS’) and an exploratory PCA, which produced a six-factor structure (the ‘exploratory 6-FS’). Thus, it was possible to examine whether NCD in non-standard datasets such as the present dataset would produce NCSs that follow the standard FS rather than the local (exploratory or confirmatory ) FSs. If that would be the case, NCD would show greater generalizability of its modularity than PCA. As a null hypothesis, we expected the generalizability of NCD to be the same or worse than that of PCA. Thus, we expected NCSs to show a better match with exploratory and confirmatory FSs from the present dataset than with the standard FS, since the latter is derived from a different (norm) dataset. Network graphs of the ‘winning’ network cluster decompositions were produced at both item- and facet levels. This provided the first description of human personality structure in terms of an optimized network of mutually dependent personality traits. Such a ‘Personality Web’ shows certain paths (sequences of traits) that represent an array of routes (“highways”) that subjects may take during the course of personality development. We identified network nodes and modules that are potentially important for personality development in terms of their singular attributes and position within the personality network.

## Materials and Methods

### Ethics Statement

All subjects in this study provided both verbal and written informed consent and were aware that their personality rating scores were to be used for research purposes. This procedure was approved by the Ethics Committee of the Department of Psychology of the University of Amsterdam, under project number 2008-PN-427. No research was conducted outside our country of residence (The Netherlands) or outside of the context of the institutions that contributed to this study (see affiliations).

### Subjects

A group of 434 healthy Dutch psychology students was selected for this study. The only inclusion criterion was the ability to sustain an interview of about 40 minutes. Exclusion criteria were signs of psychopathology as defined by DSM-IV-TR (as assessed by SCL-90) and a native language other than Dutch. Male to female ratio was 28.4% versus 71.6%, mean age was 20.6 years (SD 5.39, range 17–61).

### Psychometrics

All subjects completed the NEO-PI-R self-rating scale [15]. This scale consists of 240 items, which are scored on a six-point Likert scale (i.e. 0 to 6). The NEO-PI-R identifies 5 factors: neuroticism (N), extraversion (E), openness (O), agreeableness (A), and conscientiousness (C). Factors consists of 6 facets (e.g. n1, n2, n3, n4, n5 and n6) and each facet contains 8 items of the questionnaire.

### Factor analysis

**Figure 1:**
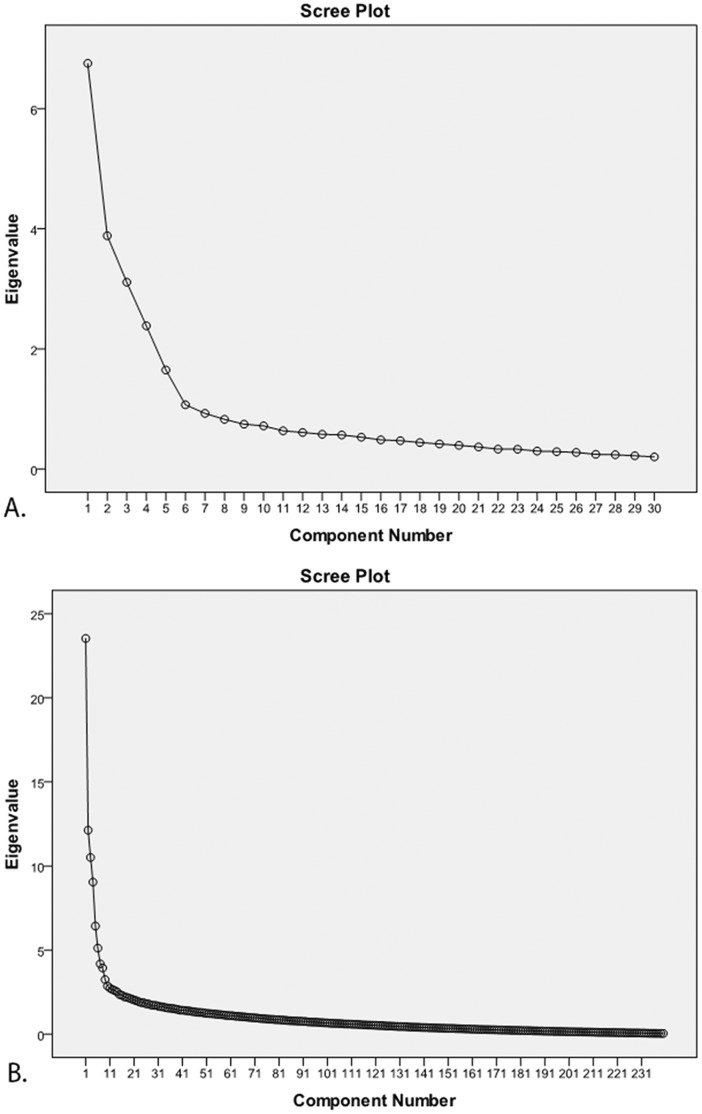
Scree-plots of facet level (A) and item level (B) principal component analyses. **A.** At facet level, a 6-factor structure is suggested by the screeplot. **B.** At item level, a 10-factor structure is found without any resemblance to either a 5-factor structure or a 30-facet structure. Such item level decompositions are known to be unreliable in smaller-than-standard datasets such as the present dataset. Hence, both 5-factor (‘confirmatory’) and 6-factor (‘exploratory’) PCAs were performed, to force item level results to more plausible solutions. The factor structures of these PCAs, rather than the 10-factor structure, served as templates in the NCS-to-FS matching procedure. See text for further details.

At facet level, exploratory and confirmatory PCA produced a 6 factor and a 5 factor decomposition, respectively (see Figure 1). To maximize comparability with standard (norm) findings, factor analyses were performed using the same specifications applied when defining the norm dataset [15]: PCA with Varimax rotation was performed in SPSS. Factors were identified at eigenvalues >1 and by inspection of the screeplot. Factor membership for items was established at item loadings >0.40.

As expected, exploratory PCA on item-scores in the present (non-standard) dataset produced implausible results: a 10-factor solution was found that, on visual inspection, showed no resemblance to either the standard 5-factor structure or any of the 30 facets of the NEO-PI-R (Table S1). Such findings are a common finding in non-standard datasets and put limits to the use of exploratory factor analysis at item level [5]. In order to force the factor structure at item level toward a more plausible solution, we performed two PCAs at item level: one involving a 5-factor structure (corresponding to the number of factors in the standard dataset) that we termed a “confirmatory” 5-factor structure and one involving an a 6-factor structure which we termed an “exploratory 6-factor structure”. Although this analysis technically involved a confirmatory 6-factor analysis, we termed it “Exploratory” because of its reference to the exploratory factor structure at facet level that indicated a 6-factor structure. Thus, three factor structures were used for facet level data: a standard (5-factor) solution, a confirmatory (5-factor) solution and an exploratory (6-factor) solution (Table 1). Similarly, three factor structures were used for item level data: the standard (5-factor) solution, a confirmatory (5 factor) solution and an “exploratory” 6-FS (Table S1). These FSs served as templates against which the NCS of the NEO-PI-R was optimized (see below).

**Table 1:** The results of standard 5-factor, confirmatory 5-factor and exploratory 6-factor PCAs of our dataset.

								Factor structure								
Type			Standard					Confirmatory					Exploratory			
FACET	N	E	O	A	C	Factor 1	Factor 2	Factor 3	Factor 4	Factor 5	Factor 1	Factor 2	Factor 3	Factor 4	Factor 5	Factor 6
n1	**1**					**.858**					**.858**					
n2	**1**					**.592**			−.570		**.588**			−.524		
n3	**1**					**.770**					**.764**					
n4	**1**					**.677**					**.679**					
n5	**1**									−.435	.404				**−.412**	
n6	**1**					**.790**					**.794**					
e1		**1**					**.711**					**.760**				
e2		**1**					**.732**					**.768**				
e3		**1**					**.551**					**.457**				
e4		**1**					**.676**					**.684**				
e5		**1**					**.466**					**.443**		−.410		
e6		**1**					**.611**					**.578**		.483		
o1			**1**					**.689**					**.670**			
o2			**1**					**.733**					**.701**			
o3			**1**					**.670**					**.488**			
o4			**1**					**.401**				.502				**.527**
o5			**1**					**.691**					**.690**			
o6			**1**					**.504**								**.630**
a1				**1**					**.559**			.463		**.514**		
a2				**1**					**.750**					**.768**		
a3				**1**			.514		**.539**					**.501**		
a4				**1**					**.719**					**.721**		
a5				**1**					**.681**					**.692**		
a6				**1**					**.524**				**.721**			
c1					**1**					**.675**					**.672**	
c2					**1**					**.749**					**.754**	
c3					**1**					**.645**					**.640**	
c4					**1**					**.718**					**.730**	
c5					**1**					**.771**					**.770**	
c6					**1**					**.714**					**.706**	

Factor loadings >0.4 are shown. A forced-choice filter was performed on factor loadings to create the final matching templates (see Materials and Methods). The highest factor loadings determined the final factor membership of individual items or factors in the templates. These highest loadings are shown in bold.

### Network analysis

#### Network graphs

Network graphs were constructed at both item and facet levels from symmetrical univariate correlation matrices, with rows and column names referring to items or facets. These matrices were filled with the corresponding correlation coefficients (r) and transformed into undirected and weighted network graphs by means of NodeXL [22]. In these networks, nodes (vertices) refer to items or facets, links to significant correlations between the nodes, and the weights of the links to the corresponding correlation coefficients.

#### Network cluster detection

In order to identify network clusters, we used the Wakita-Tsurumi NCD algorithm integrated within NodeXL [23]. This algorithm is a more efficient variant of the Clauset Newman Moore (CNM) algorithm that finds community structure (“cliquishness”) of nodes within networks in a bottom-up manner, “greedily” optimizing on the modularity of the network graph [24]. The optimal NCS is found by iteratively merging individual pairs of nodes into clusters in a balanced way (and these clusters into superclusters and so on), until maximum ‘modularity’ is reached. Modularity is defined as a quality measure that describes the extent to which “internal” connectivity measures of network clusters are higher than those of the remaining “external” network. Groups of nodes that share a maximum of connections amongst themselves rather than with their surroundings are high modularity clusters (i.e. high-quality clusters). The Wakita-Tsurumi algorithm implemented in NodeXL deviates from the original version by not including the “heuristics” that help network communities grow in a balanced way (see text). For further details, see [23].

### Matching network community structure to factor structure

In contrast to computer networks or the internet, links in correlational network graphs are present with a certain probability, or ‘significance’. The p-score of a correlation (or network link) expresses the probability that the correlation is unjustified (i.e. the link is not there). Hence, the smaller p, the higher the chance of a connection being present. Hence, the identification of an optimal NCS in correlational network graphs (such as a graph of the NEO-PI-R dataset) involves the identification of a level of probability p for the significance of a link at which the NCS of the network is optimal. Until now, a definitive way of defining a p value at which an optimal NCS is obtained has been lacking from the international literature. Here, we describe a procedure by which the global threshold of the facet- and item level personality networks is gradually raised (i.e. the networks are pruned in a link-by-link fashion), in the order of increasing r (correlation coefficient, which is directly linked to p), until an optimal match is found between NCS and different template FSs. This “incremental pruning” technique gradually removed lowly significant links from the network. After removal of each link, NCD was applied and the resulting subgraph and corresponding network cluster decomposition was saved for further analysis. At facet level, this procedure resulted in a total of 420 network cluster decompositions ((30*30)/2 -30 links). At item level, a total of 28560 network cluster decompositions ((240*240)/2 -240 links) were produced. These NCSs were matched against the factor contents of the three alternative FSs produced at item- and facet levels (standard 5-FS, confirmatory 5-FS and exploratory 6-FS), which served as matching ‘templates’. The matching of network cluster composition with factor structures involved the comparison of the ‘factor membership’ and ‘network cluster membership’ of each individual facet and item of the NEO-PI-R rating scale. Factor membership was represented as a two-dimensional binary matrix (i.e. factor number×facet number, and factor number×item number), which was filled with ones (1) for membership and zeroes (0) for non-membership. A similar matrix was made for network cluster membership (cluster number×facet number, or cluster number×item number). This was done for all individual subgraphs derived from the incremental pruning stage. Mismatches between factor and cluster structures were identified by subtraction of the binary membership matrices of factor structures and network cluster structures for all subgraphs and corresponding network clusters derived from incremental pruning, resulting in a cluster-to-factor mismatch (dissimilarity) measure for each subgraph and corresponding set of clusters. Since factors and clusters could differ in their respective sizes, the level of mismatch could differ between comparisons on this account. To prevent unreliable mismatch scores as a result of differences in factor or cluster sizes, we normalized the mismatch scores with respect to these size differences, by taking the absolute mismatch score for each cluster-to-factor comparison and dividing it by the maximum possible mismatch score for that comparison (i.e. factor size+cluster size).

Factor solutions allow the same items or facets to load on multiple factors (Table 1). Hence, an item or a facet can be a member of different factors. In contrast, the Wakita-Tsurumi NCD algorithm performs a forced-choice assignment of items and facets to their network clusters (a common feature of most NCD algorithms). This difference in classification produces additional levels of mismatch if unaccounted for. Factor loadings were therefore subjected to a similar forced-choice filter in order to create the templates that were used for matching FS to NCS. If items or facets showed multiple factor loadings, the facet or item that showed the highest factor loading determined its factor membership in the template.

In some cases, the matching procedure could result in more than one solution with equally low cluster-to-factor mismatch scores at different thresholds for the significance of a link (e.g. Facet Level_SOLUTION1 and Facet Level_SOLUTION2). If multiple community structures were found that showed an equal lowest levels of mismatch, a winning NCS was identified by selecting the NCS that explained the greatest amount of variance in the corresponding factor scores. To this end, network cluster scores were calculated by summing facet or item-scores and dividing the result by the total number of facets in the network cluster. Next, the correlation coefficients of significant correlations (p<0.01) between cluster and factor scores were squared (r^2^) and summed to produce a measure of the total amount of variance in factor scores, as explained by network cluster scores.

### Calculating network metrics

For item-, facet- and cluster level networks, the following network metrics were calculated for each node [21]: degree, betweenness centrality, closeness centrality, eigenvector centrality, pagerank and clustering coefficient. Overall network metrics were calculated that included mean values of these parameters, modularity, mean average pathlength and graph density measures. Finally, a measure was calculated for item- and facet level networks that expresses their degree of ‘small-worldness’ [25]:
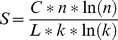
where C, n, L, and k are the mean clustering coefficient, number of nodes, mean shortest path length, and mean node degree of the network, respectively. This measure compares the ratio of mean clustering coefficient and mean shortest path length between the empirically derived (NEO-PI-R) network and a randomly connected graph of the same size. Since small-world networks are non-randomly connected (with large mean clustering coefficients and short mean pathlengths) this ratio is larger for small-world network than for randomly connected graphs of the same size. Hence, if S>1, the graph can be considered to have small-world properties.

## Results

### Results of factor analyses

Table 1 shows the results of confirmatory (5 factor) and exploratory factor analyses of our dataset. See Figure 1 for the corresponding screeplots.

At facet level, exploratory factor analysis showed a 6-factor structure that deviated from the standard 5-factor structure, although the standard structure could still be largely recognized (Figure 1A, Table 1). The confirmatory 5-factor analysis produced a decomposition that showed a high degree of resemblance with the 5-factor standard structure (Table 1). The Kaiser-Meyer-Olkin (KMO) score of the dataset was 0.864, indicating very good sampling adequacy. Bartlett's test of sphericity showed a Chi square of 6221.5, df = 435, p∼0, indicating high sphericity of the dataset. The exploratory 6-factor analysis explained 62.8% of total variance in the facet scores, whereas the confirmatory 5-factor analysis explained 59.2% of total variance.

As expected, PCA at item level produced erroneous or weak results (see introduction, M&M). Exploratory PCA showed a 10-factor structure without any resemblance to either a 5-factor structure or a 30-facet structure (Figure 1B). A confirmatory 5-factor analysis produced a factor structure in which the standard 5-factor structure could be recognized, but only if low factor loadings were allowed (i.e. >0.13; Table S1). The “Exploratory” 6-factor PCA showed similar results, with a further degradation of the norm structure due to low item loadings (Table S1). At item level, the Kaiser-Meyer-Olkin (KMO) score of the dataset was 0.750, indicating good sampling adequacy. Bartlett's test of sphericity showed a Chi square of 58845.8, df = 28680, p∼0, indicating high sphericity of the dataset. The confirmatory 5-factor analysis explained 25.7% of total variance in the item scores, whereas the exploratory 6-factor analysis explained 27.8% of total variance.

### Matching FS to network cluster structure: facet level

**Figure 2:**
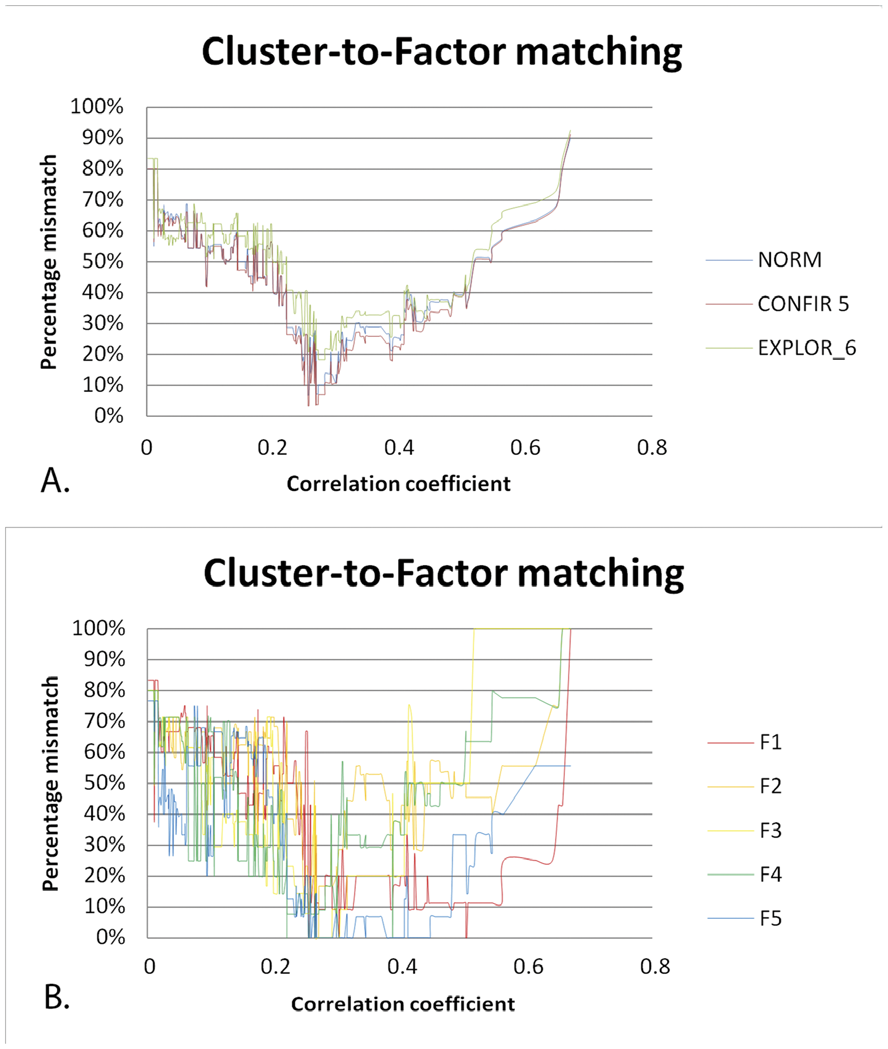
Results of the network community structure to factor structure matching procedure at facet level. **A.** Results of the NCS-to-FS matching procedure for standard, confirmatory and exploratory FSs. X-axis shows the correlation coefficient r as a threshold for significance of a link in the network graph (as r increases to the right, more links are pruned from the network). Y-axis shows normalized dissimilarity (mismatch) scores. Blue: standard 5-FS, red: confirmatory 5-FS, green: exploratory 6-FS. The confirmatory 5-FS shows the best match with NCS at r = 0.271, p = 4.89E-09. For details, see text and Tables 1 and 2. **B.** Results of the NCS-to-FS matching procedure for the specific case of the winning confirmatory 5-FS (red line in Fig A), with a subspecification of the matching results per factor. F1–F5: confirmatory factors resembling Neuroticism, Extraversion, Openness, Agreeableness and Conscientiousness, respectively. For details, see text and Tables 1 and 2.

Figure 2A shows the results of matching the three different FSs (standard 5-FS, confirmatory 5-FS and exploratory 6-FS) to the network cluster structures of the full range of subgraphs produced by incremental pruning of the facet level network. The correlation matrix of facet scores contained 434 correlations from r = 0 to r = 0.671. The closest match of FS with NCS was found with the confirmatory 5-FS, with an average mismatch per factor of only 3.76% (see below for a further specification). The standard 5-FS also showed a quite reasonable match, with average mismatch scores per cluster of 7.1% and neuroticism showing the greatest deviance (20%). The exploratory 6-FS showed the highest mismatch scores (18.31%). Paired T-tests showed that NCS was significantly closer to the confirmatory 5-FS across the entire range of subgraphs than standard 5-FS (mean difference = 1.4%, T = 20.51, df = 433, p∼0) and the exploratory 6-FS (mean difference = 5.7%, T = −24.18, df = 433, p∼0). Thus, both global and local best fits were found for the confirmatory 5-FS. Across the entire range of facet level subgraphs, no clear match was found with the sixth factor of the exploratory 6-FS (33% match at best), see Table 2.

**Table 2:** Table showing the quantitative results of the cluster-to-factor matching procedure at facet level.

	Cluster to factor matching			BEST OVERALL MATCH			BEST MATCH PER FACTOR	
	Facet Level		r	p	% mismatch	r	p	% mismatch
		N	0.271	4.89E-09	20.0%	0.215	3.07E-06	9.1%
		E	0.271	4.89E-09	0.0%	0.271	4.89E-09	0.0%
	STANDARD (5)	O	0.271	4.89E-09	0.0%	0.271	4.89E-09	0.0%
		A	0.271	4.89E-09	7.7%	-0.265	1.10E-08	0.0%
		C	0.271	4.89E-09	7.7%	-0.299	1.04E-10	0.0%
		F1	0.271	4.89E-09	11.1%	0.215	3.07E-06	0.0%
		F2	0.271	4.89E-09	0.0%	0.271	4.89E-09	0.0%
Factor structure	CONFIRMATORY (5)	F3	0.271	4.89E-09	0.0%	0.271	4.89E-09	0.0%
		F4	0.271	4.89E-09	7.7%	−0.265	1.10E-08	0.0%
		F5	0.271	4.89E-09	0.0%	0.271	4.89E-09	0.0%
		F1	0.282	1.18E-09	9.1%	0.303	5.53E-11	0.0%
		F2	0.282	1.18E-09	16.7%	0.282	1.18E-09	16.7%
	EXPLORATORY (6)	F3	0.282	1.18E-09	9.1%	0.306	3.67E-11	0.0%
		F4	0.282	1.18E-09	0.0%	0.282	1.18E-09	0.0%
		F5	0.282	1.18E-09	0.0%	0.282	1.18E-09	0.0%
		F6	0.282	1.18E-09	75.0%	0.266	9.05E-09	33.3%

Standard, confirmatory, exploratory: type of factor analysis. N, E, O, A, C: NEUROTICISM, EXTRAVERSION, OPENNESS, AGREEABLENESS, CONSCIENTIOUSNESS. F1–F6: factors of the corresponding factor analysis. F1–F6 correspond to N, E, O, A,C, and a 6th factor that is the product of the exploratory factor analysis of the current dataset. Best overall match: best match between overall cluster contents and overall FS. r and p: r and p values at which the best overall match is found. Best cluster-to-factor match: best match between individual factors and network clusters, which may occur at different r and p-values per cluster. % mismatch denotes the percentage of normalized mismatch between factor and network cluster contents. Note that FSs and NCSs at facet level generally show high degrees of correspondence (96.2%), with a best match occurring with the confirmatory 5-FS at r = 0.271, p = 4.89E-09.

**Figure 3:**
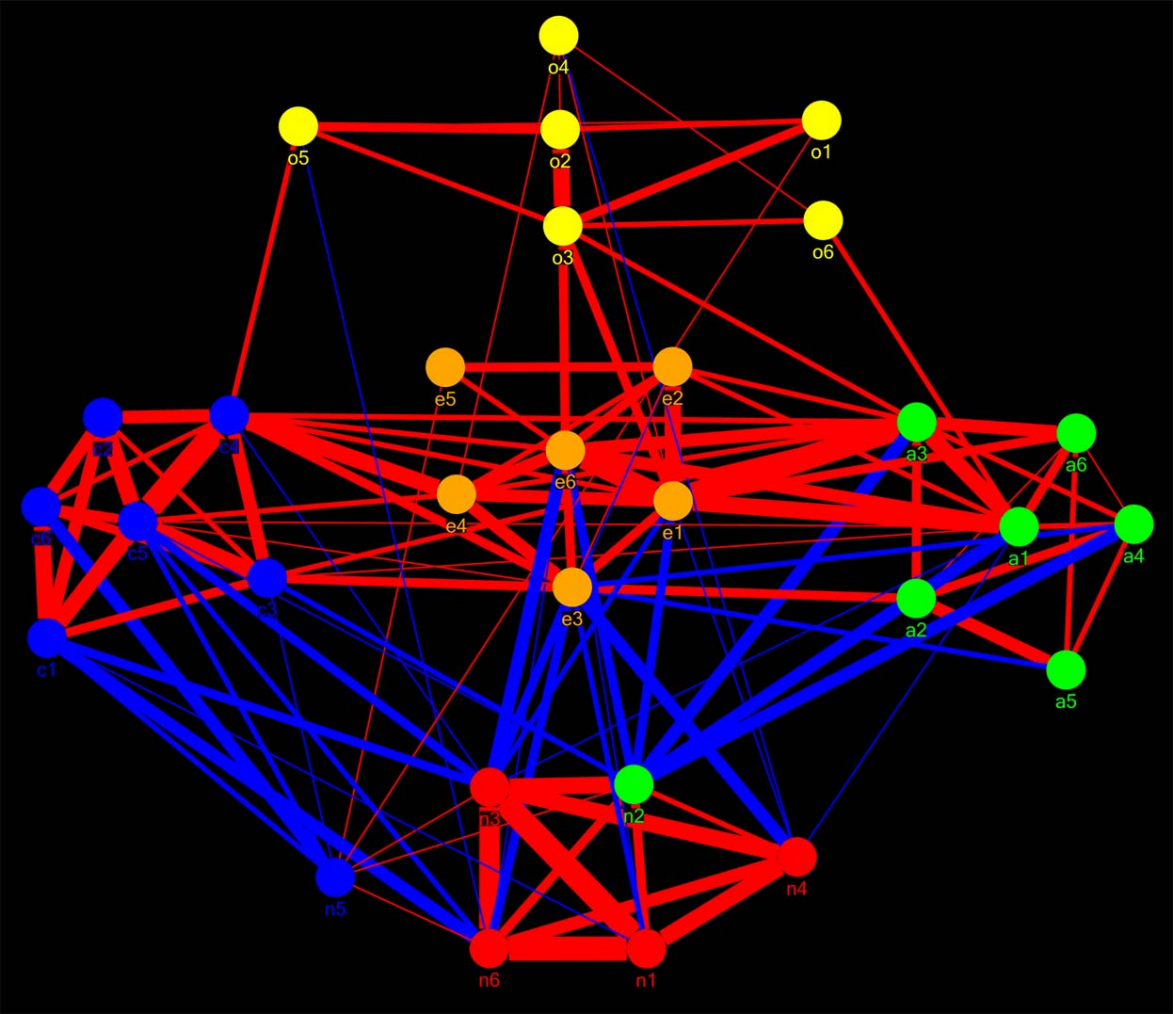
The Personality Web at facet level: network graph of correlational relationships between the 30 facets of the NEO-PI-R. The community structure of this graph has an overall best fit with the confirmatory 5-FS, occurring at r>0.271, p<4.89 E-09. See Table 2 for significances and correlation coefficients. Node = facet, link = significant correlation. Red links: positive correlations. Blue links: negative correlations. The thickness of the links represents the strength of the correlation. For further information, see Table 4B. n = neuroticism, e = extraversion, o = openness, a = agreeableness, c = conscientiousness. Numbers refer to facet number. Nodes are positioned in clusters according to their factor membership (standard 5-FS). The color of nodes denotes their network cluster membership. Only two facets show a mismatch with the standard 5-FS (n5 and n2). Both mismatches involve the neuroticism dimension depicted below in red. These facets have strong correlations with facets from the conscientiousness cluster (blue) and the agreeableness cluster (green), as can be observed by the thickness of the corresponding links. As a result, n5 is “drawn” into the conscientiousness cluster and n2 into the agreeableness cluster.

Figure 2B shows the degree of mismatch between network clusters and factors for the specific case of the (winning) confirmatory 5-FS. Two different NCSs were found at different global thresholds that showed the same global minimum of mismatch with the confirmatory 5-FS. These were termed “Facet Level_SOLUTION1” and “Facet Level_SOLUTION2”. Facet Level_SOLUTION1 was stable for 3 consecutively pruned links for (r = 0.255 to 0.256, p = 3.60 E-08 to 3.08 E-08) and Facet Level_SOLUTION2 was stable for 8 consecutively pruned links (r = 0.268 to 0.271, p = 7.25 E-09 to 4.89 E-09). Facet Level_SOLUTION2 showed a trend of explaining more variance in the factor scores of the confirmatory (5) factor solution than Facet Level_SOLUTION1 (T = 1.83, df = 24, p = 0.08, two samples T-test). Hence, this solution was chosen as the winning solution. The largest amount of explained variance was found at r = 0.271, p = 4.89 E-09. At this threshold, three network clusters showed a complete match with their corresponding factors (cluster 2 & EXTRAVERSION, cluster 3 and OPENNESS, and cluster 5 and CONSCIENTIOUSNESS), one cluster showed a 7% mismatch (cluster 1 and NEUROTICISM) and another an 11% mismatch (cluster 4 and AGREEABLENESS), see Table 2. The cluster scores of these network clusters explained an average of 96.2% of the variance in the factor scores of the confirmatory 5-FS. Additionally, these cluster scores explained 69.6% of total variance observed in the facet level dataset (15.0%, 16.6%, 11.8%, 11.7% and 14.5% for cluster 1, 2, 3, 4, and 5, respectively). Hence, more variance is explained by NCD than by PCA (either in confirmatory or exploratory analyses). Figure 3 shows the facet level network graph.

When the requirement for an overall match between NCS and FS was dropped, some individual factors showed a better match with individual clusters at various thresholds (see Table 2). This may indicate that individual network clusters require their own global threshold for optimal representation. However, the thresholds for these individual best matches all clustered quite tightly around the threshold for the best overall match (Table 2), suggesting that the overall best match is a good estimation of the individual best matches.

### Matching FS to network cluster structure: item level

**Figure 4:**
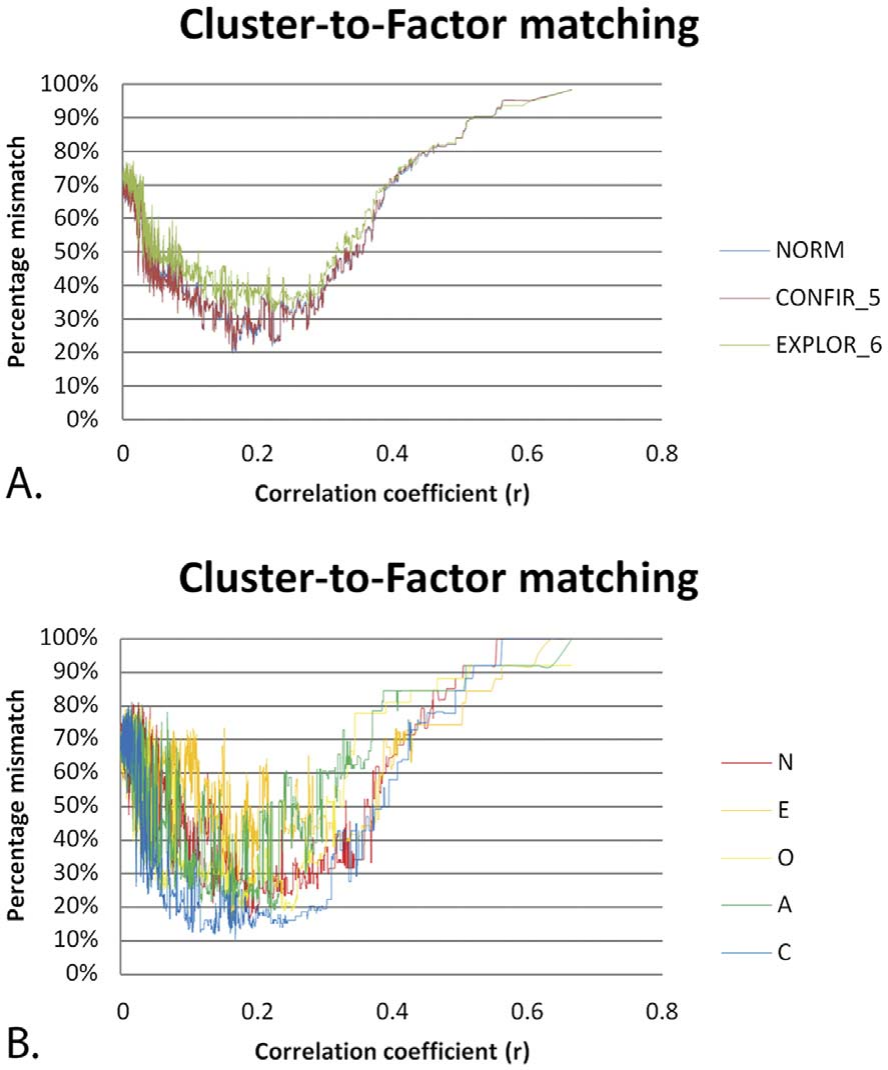
Results of the network community structure to factor structure matching procedure at item level. **A.** Results of the NCS-to-FS matching procedure for standard, confirmatory and exploratory factor analyses. Same specifications as in Figure 2 apply. Minimal dissimilarity (mismatch) is found with the standard 5-FS at r = 0.164, p = 3.08E-04. For details, see text and Tables 1 and 3. **B.** Results of the NCS-to-FS matching procedure for the specific case of the winning standard 5-FS (blue line in Figure A), with a subspecification of the matching results per factor. N, E, O, A,C: NEUROTICISM, EXTRAVERSION, OPENNESS, AGREEABLENESS and CONSCIENTIOUSNESS. Different factors show various degrees of matching with NCSs. For details, see text and Tables 1 and 3.

Figure 4A shows the results of matching the three different FSs (standard 5-FS, confirmatory 5-FS and exploratory 6-FS) to the network cluster structures of the full range of subgraphs produced by incremental pruning of the item level network. The correlation matrix of item scores contained 28680 correlations from r = 0 to r = 0.759. The closest match of NCS with FS was found with the standard 5-FS, with an average mismatch per factor of 19.8%. This was closely followed by the confirmatory 5-FS, with an average mismatch per factor of 20.6%. The best match with the exploratory 6-FS was more problematic, with an average mismatch per factor of 28.9%. Paired T-tests showed that NCS was significantly closer to the confirmatory 5-FS across the full range of subgraphs than standard (mean difference = 0.6%, T = 114.7, df = 28678, p∼0) or exploratory factor solutions (mean difference = 5.8%,T = −348.04, df = 28678, p∼0). Despite such global fits, the best local fit of NCS was found with the standard factor structure. Across the full range of item level subgraphs, no clear match was found with the sixth factor of the exploratory 6-FS (17.3% match at best), see Table 3.

**Table 3:** Table showing the quantitative results of the cluster-to-factor matching procedure at item level.

	Cluster to factor matching			BEST OVERALL MATCH			BEST MATCH PER FACTOR	
	Item Level		r	p	% mismatch	r	p	% mismatch
		N	0.164	3.08E-04	27.1%	0.190	3.38E-05	16.7%
		E	0.164	3.08E-04	16.7%	0.164	3.04E-04	16.7%
	STANDARD (5)	O	0.164	3.08E-04	16.3%	-0.166	2.57E-04	14.9%
		A	0.164	3.08E-04	20.4%	0.158	4.64E-04	17.9%
		C	0.164	3.08E-04	18.4%	0.170	1.86E-04	10.4%
		F1	−0.169	2.03E-04	28.4%	0.220	1.92E-06	17.5%
		F2	−0.169	2.03E-04	17.9%	0.164	3.04E-04	16.7%
Factor structure	CONFIRMATORY (5)	F3	−0.169	2.03E-04	19.5%	0.166	2.57E-04	14.9%
		F4	−0.169	2.03E-04	20.8%	0.158	4.64E-04	17.9%
		F5	−0.169	2.03E-04	16.4%	−0.140	1.72E-03	13.3%
		F1	−0.169	2.03E-04	28.4%	0.220	1.92E-06	17.5%
		F2	−0.169	2.03E-04	22.1%	0.164	3.04E-04	20.8%
	EXPLORATORY (6)	F3	−0.169	2.03E-04	13.5%	0.164	3.04E-04	13.2%
		F4	−0.169	2.03E-04	27.3%	0.159	4.59E-04	22.4%
		F5	−0.169	2.03E-04	16.4%	0.140	1.72E-03	13.3%
		F6	0.221	1.71E-06	78.9%	0.148	9.73E-04	54.5%

Same specifications apply as in Table 2. Note that item level network cluster structures generally show a lesser degree of matching with the (facet level) FSs, with a best overall match (80.0%) occurring with the standard 5-FS at r = 0.164, p = 3.08E-04. Individual cluster-to-factor matchings showed better fits, but no match was larger than 90%. See discussion for further details.

**Figure 5:**
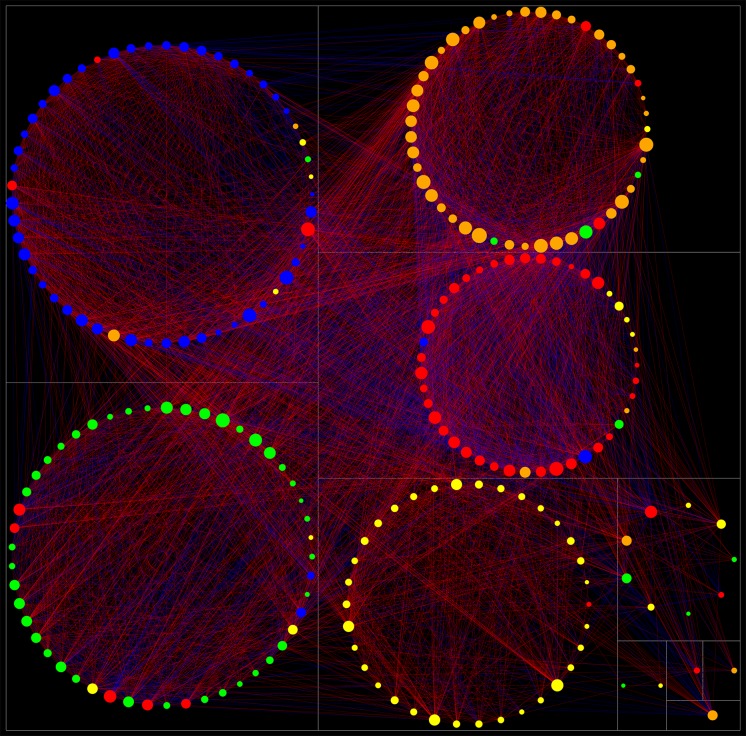
The Personality Web at item level: network graph of correlational relationships between the 240 items of the NEO-PI-R. The community structure of this graph has an overall best fit with the standard 5-FS, occurring at r = 0.164, p = 3.08E-04. Node = item, link = significant correlation. Red links: positive correlations. Blue links: negative correlations. Node size = degree (larger nodes are bigger hubs, scale = 1 to 10). For further information, see supporting information (Table S2). The color of the items refers to their standard factor membership, i.e. red: Neuroticism, orange: Extraversion, yellow: Openness, green: Agreeableness, blue: Conscientiousness. Items are grouped together according to their network cluster membership, and clusters are depicted as circles. Clusters are grouped according to size. Five clusters are found that show maximum correspondence to standard factors (Table 3,4). A small 6th factor is found (right in the graph), consisting of 9 items belonging to extraversion (1 item), openness (3 items), agreeableness (3 item) and neuroticism (2 items). Four isolates (bottom-right corner) consisting of 1 or 2 items were discarded from further analysis. Most of the top 10% hubs are located in the extraversion and neuroticism clusters.

Figure 4B shows the degree of mismatch between factors and network clusters for the specific case of the (winning) standard 5-FS. Across the full range of subgraphs, only one community structure (Item Level_SOLUTION1) was found that showed the best overall match with this FS. This solution was stable across a range of 19 consecutively pruned links (corresponding to r = 0.1635–0.1638, p = 3.13E-04−3.08E-04). The clusters of this community structure explained an average of 80% of the variance in their most corresponding factors. Overall, the item level cluster scores explained 33.2% of total variance observed in the item scores (i.e. 6.4%, 3.6%, 8.0%, 4.5%, 6.4% and 4.3%, for cluster 1, 2, 3, 4, 5 and 6 respectively). This is a larger amount of variance than explained by item level factor analysis (24.4%). Figure 5 shows the corresponding item level network graph.

When the requirement for an overall match between NCS and FS was dropped, better matches were found for individual cluster-to-factor comparisons (up to 10.43% mismatch for network cluster 5 with conscientiousness of the standard factor solution), see Table 3. The thresholds for these individual matches clustered around the threshold for the best overall match (Table 3), again suggesting that the global best match is a good estimation of the individual best matches.

**Figure 6:**
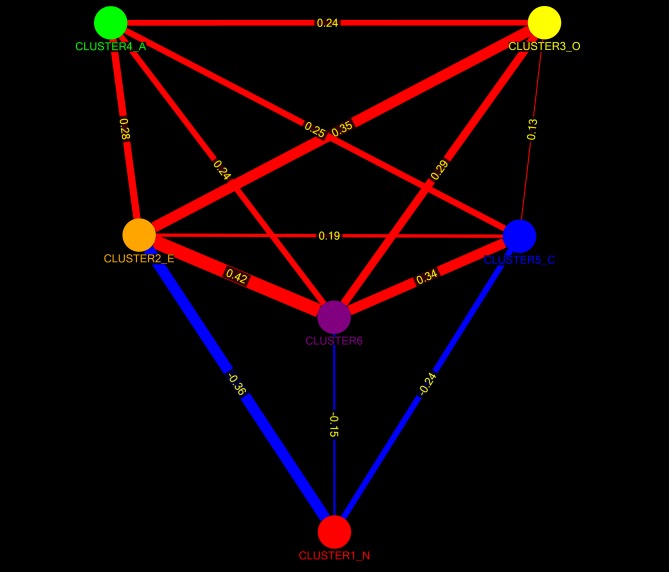
The Personality Web at cluster level: network graph showing correlations between cluster scores calculated using the network community structure from **Figure 5**. CLUSTER1_N, CLUSTER2_E, CLUSTER3_O, CLUSTER4_A, CLUSTER5_C: clusters showing maximum correspondence with standard clusters of Neuroticism, Extraversion, Openness, Agreeableness and Conscientiousness, respectively. CLUSTER6: newly found sixth factor (see Figure 5, Table 4 and Discussion). Network graph is shown at p<0.01 corrected for multiple comparisons. The thickness of the links represents the strength of the correlation coefficient. Correlation coefficients are shown alongside the links. Red: positive correlations, blue: negative correlations. For further information, see Table 4A. CLUSTER1_N, the Neuroticism analogue, has only negative (inhibitory) influences on the remaining network structure, which shows only positive interrelations. Personality Webs such as these may represent developmental structures. CLUSTER2_E (Extraversion analog), CLUSTER5_C (Conscientiousness analog) and the 6^th^ cluster form an intermediate structure in between CLUSTER1_N (Neuroticism analog) and CLUSTER4_A and CLUSTER3_O (the Agreeableness and Openness analogs). This suggests that the negative influence of Neuroticism on Agreeableness and Openness scores, as observed in personality disorders, is mediated by this intermediate level (see discussion for further details).

Network clusters at item level immediately produced large-scale clusters showing good correspondence with standard factors. No evidence was found for an intermediate facet level at either higher or lower thresholds. The NCS that showed an optimal match with the standard 5-FS involved a six-cluster network structure. The sixth cluster contained a total of 9 items. These involved one extraversion item, two neuroticism items (with negative correlations with the other items within the cluster), three openness items and three agreeableness items (Table S2, Figure 5). Some individual items (‘isolates’) were not classified into separate clusters and were discarded from further cluster level analysis. Cluster scores were calculated for all six clusters and a correlation matrix was generated at p<0.01, corrected for multiple comparisons. Figure 6 shows the corresponding cluster level network graph.

### Network metrics

Table 4A and 4B show the content of network clusters for the winning NCSs at facet- and cluster level, along with corresponding network metrics. Similar data for the item level network are given as supporting information (Table S2). Nodes with central importance for information transfer through the Personality Web were identified by means of their degree (hubs) and betweenness centralities (see discussion). At item level, the top 10% hubs were located mostly in the extraversion cluster. Additionally, the neuroticism and (to a lesser degree) conscientiousness clusters were rich in hub nodes (Figure 5). A similar distribution across clusters was seen for the 10^th^ percentile of nodes with highest betweenness centralities. The openness cluster was particularly devoid of singular nodes. Table 5 shows the overall network metrics for these three graphs. With respect to network topology, a value of S = 2.58 was found for the facet level network and a value of S = 1.94 for the item level network, indicating that the personality network at both levels is characterized by a small-world structure.

**Table 4:** Cluster contents and node-specific network metrics for the network graphs described in Figures 3 and 6.

Network Cluster	Node	Facets	Degree	Betweenness Centrality	Closeness Centrality	Eigenvector Centrality	PageRank	Clustering Coefficient
1	CLUSTER1_N	See supp. Inf.	3	0.000	0.143	0.125	0.730	1.000
2	CLUSTER2_E	See supp. Inf.	5	0.667	0.200	0.184	1.138	0.800
3	CLUSTER3_O	See supp. Inf.	4	0.000	0.167	0.161	0.927	1.000
4	CLUSTER4_A	See supp. Inf.	4	0.000	0.167	0.161	0.927	1.000
5	CLUSTER5_C	See supp. Inf.	5	0.667	0.200	0.184	1.138	0.800
6	CLUSTER6	See supp. Inf.	5	0.667	0.200	0.184	1.138	0.800
A.								

Network Cluster	Node	Facet	Degree	Betweenness Centrality	Closeness Centrality	Eigenvector Centrality	PageRank	Clustering Coefficient
1	n1	n1	7	2.090	0.019	0.034	0.825	0.714
1	n3	n3	13	15.507	0.022	0.060	1.419	0.526
1	n4	n4	8	7.685	0.019	0.037	0.942	0.571
1	n6	n6	10	13.668	0.020	0.043	1.147	0.511
2	e1	e1	13	25.286	0.022	0.055	1.464	0.449
2	e2	e2	8	6.439	0.019	0.038	0.951	0.607
2	e3	e3	12	28.507	0.021	0.050	1.350	0.409
2	e4	e4	8	8.127	0.020	0.037	0.946	0.571
2	e5	e5	3	0.983	0.016	0.013	0.450	0.333
2	e6	e6	14	40.807	0.023	0.057	1.584	0.396
3	o1	o1	4	4.023	0.015	0.008	0.615	0.500
3	o2	o2	5	5.130	0.016	0.011	0.734	0.400
3	o3	o3	7	16.556	0.018	0.020	0.952	0.333
3	o4	o4	5	5.775	0.016	0.015	0.708	0.200
3	o5	o5	5	6.744	0.017	0.013	0.715	0.300
3	o6	o6	3	1.452	0.015	0.009	0.482	0.000
4	a1	a1	13	35.185	0.021	0.053	1.475	0.397
4	a2	a2	7	5.856	0.018	0.026	0.869	0.667
4	a3	a3	12	24.946	0.022	0.049	1.363	0.455
4	a4	a4	7	3.867	0.017	0.027	0.867	0.571
4	a5	a5	4	0.963	0.015	0.013	0.565	0.667
4	a6	a6	6	2.883	0.016	0.022	0.770	0.667
4	n2	n2	13	22.977	0.022	0.057	1.432	0.449
5	c1	c1	9	6.755	0.018	0.036	1.048	0.583
5	c2	c2	5	0.000	0.016	0.020	0.644	1.000
5	c3	c3	10	17.680	0.020	0.040	1.150	0.511
5	c4	c4	12	32.751	0.021	0.049	1.356	0.409
5	c5	c5	12	19.192	0.021	0.050	1.338	0.470
5	c6	c6	6	0.850	0.016	0.023	0.750	0.867
5	n5	n5	9	22.318	0.019	0.033	1.090	0.417
B.								

**A:** Cluster level network graph shown in Figure 6.

**B.** Facet level network graph shown in Figure 3. For item-level data (Figure 5), see supporting information (Table S2).

**Table 5:** Overall network metrics for the ‘winning’ network graphs described in this paper.

Metric	A. Fig. 3	B. Fig. 5	C. Fig. 6.
Level of detail	FACET LEVEL	ITEM LEVEL	CLUSTER LEVEL
Vertices	30.0000	240.0000	6.0000
Unique Edges	125.0000	5738.0000	13.0000
Edges With Duplicates	0.0000	0.0000	0.0000
Total Edges	125.0000	5738.0000	13.0000
Self-Loops	0.0000	0.0000	0.0000
Connected Components	1.0000	1.0000	1.0000
Single-Vertex Connected Components	0.0000	0.0000	0.0000
Maximum Vertices in a Connected Component	30.0000	240.0000	6.0000
Maximum Edges in a Connected Component	125.0000	5738.0000	13.0000
Maximum Geodesic Distance (Diameter)	4.0000	4.0000	2.0000
Average Geodesic Distance	1.8222	1.8789	0.9444
Graph Density	0.2874	0.2001	0.8667
Minimum Degree	3.0000	1.0000	3.0000
Maximum Degree	14.0000	106.0000	5.0000
Average Degree	8.3333	45.4667	4.3333
Median Degree	8.0000	41.5000	4.5000
Minimum Betweenness Centrality	0.0000	0.0000	0.0000
Maximum Betweenness Centrality	40.8066	590.6442	0.6667
Average Betweenness Centrality	12.8333	109.4292	0.3333
Median Betweenness Centrality	7.2200	79.6211	0.3333
Minimum Closeness Centrality	0.0152	0.0014	0.1429
Maximum Closeness Centrality	0.0227	0.0027	0.2000
Average Closeness Centrality	0.0186	0.0022	0.1794
Median Closeness Centrality	0.0185	0.0022	0.1833
Minimum Eigenvector Centrality	0.0079	0.0001	0.1248
Maximum Eigenvector Centrality	0.0598	0.0109	0.1842
Average Eigenvector Centrality	0.0333	0.0042	0.1667
Median Eigenvector Centrality	0.0349	0.0037	0.1727
Minimum PageRank	0.4502	0.1684	0.7304
Maximum PageRank	1.5840	2.0419	1.1380
Average PageRank	1.0000	1.0000	0.9999
Median PageRank	0.9494	0.9502	1.0328
Minimum Clustering Coefficient	0.0000	0.0000	0.8000
Maximum Clustering Coefficient	1.0000	1.0000	1.0000
Average Clustering Coefficient	0.4983	0.4818	0.9000
Median Clustering Coefficient	0.4848	0.4763	0.9000
Small-worldness	0.2735	0.2564	N/A

**A.** Facet level network graph matching with the confirmatory 5-FS of the present dataset, see Figure 3.

**B.** Item level network graph matching with the standard 5-FS of the NEO-PI-R standard dataset, see Figure 5.

**C.** Cluster level network graph generated from correlations between cluster scores, see Figure 6. The facet- and item level network graphs display a small-world topology.

## Discussion

The current study directly compared the result of network community detection (NCD) and principal component analysis (PCA) in a dataset of personality scores (NEO-PI-R). Network community structure (NCS) was matched to factor structure (FS) while gradually raising the threshold for the significance of network links until NCS showed an optimal match with FS. Our analyses show that PCA and NCD generate highly similar results, confirming the theoretical similarity between the two techniques [19], [20]. We first compared the results of PCA and NCD at facet level, since both techniques work well at this level. Next, we performed NCD at item level to examine whether NCD, as opposed to PCA, would produce plausible results at this level. Interestingly, NCD worked well at item level. Network clusters explained more variance in both facet level and item level data than corresponding FSs, suggesting that NCD provides a better summary of the data. Information could be obtained with respect to the influence of key nodes within the personality network, enabling future studies of personality development. Thus, NCS is able to overcome some of the central limitations of PCA. Below, we will first compare the results of NCA and PCA at facet and item levels. Next, we will discuss the value of the Personality Web for studies of normal personality development and personality disorders.

### Facet level

At facet level, NCD showed a best match with the confirmatory 5-FS (96.2%). A similar tight match was found with the standard FS (92.0%), which differed little from the confirmatory structure. These findings confirm our expectation that NCS would show a tight match with (standard) FSs at facet level. This match was not a matter of coincidence, given the steep decline of the mismatch curves, which clearly converged onto an optimal solution, which was stable across 8 consecutive pruning actions (Figure 2). The total amount of variance explained by network cluster scores was greater than that explained by factor scores. Hence, NCS seems to provide a better summary of the data. Both local and global bests fits were found for the confirmatory 5-FS, indicating that NCD at facet level converges toward a solution that is close to the standard 5-FS. The standard 5-FS showed a better match than the exploratory FS. This was contrary to our expectations, since the standard FS is derived from a different (norm) dataset. The mismatch with the exploratory FS was largely due to an ill match with the sixth factor, since mismatch percentages were largest for this factor. This finding indicates that PCA and NCD may produce different results for smaller factors. This can partly be explained by the forced-choice nature of the Wakita-Tsurumi algorithm. Within the context of ambiguous factor loadings of facets, the forced-choice allocation of facets to network clusters involves a chance process to some degree (i.e. correct versus incorrect classification). As a result, the cluster contents of smaller modules may show larger deviations, since misallocation is felt more severely in small clusters. For larger clusters, such effects are averaged out. Alternatively, the lack of a match with the 6^th^ (exploratory) factor may point toward a fundamental difference between the two clustering techniques (see below).

In both PCA and NCD, facets of the neuroticism dimension deviated from the standard solution and were partly redistributed across the agreeableness and conscientiousness clusters. Hence, it is possible that the neuroticism dimension in our sample of young psychology students deviated from the standard (norm) population. The redistribution of n2 and n5 facets caused an exaggerated amount of mismatch (3.8%) between NCD and PCA findings, since mismatch scores were not only found for neuroticism, but also for agreeableness and conscientiousness clusters, although these latter clusters were perfectly reproduced apart from the incorporation of neuroticism facets. NCD is therefore expected to behave even more similarly to PCA (i.e. >96.2%) when applied in larger (standard) datasets.

### Item level

In contrast to PCA, NCD at item level produced a limited set of plausible modules that showed good correspondence with the FS of the norm dataset (80%). This match was not a matter of coincidence given the dip of the mismatch curves, which clearly converged onto an optimal solution, which was stable across 19 consecutively pruned links (Figure 4). The total amount of variance explained by network cluster scores was greater than that explained by factor scores. Hence, NCD seems to surpass factor analysis in extracting modularity from item level data in non-standard samples. When compared to facet level, the overall match was lower (80%). This is most likely due to the fact that our sample deviated from the norm population both in terms of the population (college students) and sample size. A tighter match is expected if NCD would be performed in standard datasets.

NCD showed a closer global fit with confirmatory 5-FSs, confirming our expectations that NCS shows a better match with FSs derived from the same datasets. However, the winning fit was found for the standard (norm) FS, which involved a local fit. This was contrary to our expectations and introduces the possibility that NCD, when performed at item level, is able to utilize additional information that facilitates the extraction of the “true” (standard) modular structure of human personality from non-standard datasets. One explanation why NCD outperforms PCA at item level is the forced-choice nature of the Wakita-Tsurumi NCD algorithm, which dichotomizes network cluster membership. Whereas this may be problematic in smaller clusters (e.g. at facet level, see above), this has the potential of diminishing the sensitivity to chance deviations in larger clusters (e.g. at item level), since these are averaged out. However, this cannot entirely explain the better performance, since a similar forced-choice filter was applied to factor loadings to avoid differences between the results of NCD and PCA precisely for this reason. Hence, some attribute specific to NCD may be responsible for the better performance of NCD when compared to PCA. PCA first identifies the factor that explains most variance in the data, after which its effect is linearly subtracted from the data and the process repeats. Instead, NCD greedily builds modules in a bottom-up fashion, increasingly considering the global picture of the dataset. Thus, NCD may have access to a larger pool of information. Further studies are needed to examine whether NCD indeed produces results in smaller datasets that can still be generalized to the population at large.

At item level, NCD immediately identified large clusters, i.e. no intermediate (facet) level of aggregation was found. This finding is in accordance with previous factor analytic studies that found no evidence for facets as an intermediate level of aggregation in between items and factors, when adopting a bottom-up approach [18]. If these results can be reproduced with other datasets and cluster algorithms, this would add to the growing idea that facets are not separate subclusters. Instead, facets might be defined as nested hierarchies of intercorrelated items within network clusters. Such hierarchies are not detected using the current clustering algorithm, but can be examined by means of alternative cluster algorithms (e.g. [26]).

At item level, the NCS that showed an optimal likeness with (standard) 5-FS was found at a global threshold that introduced a small sixth factor next to the other five (Figure 5). The confirmatory 6-FS at item level showed a lesser fit with NCS than the standard FS. Hence, this sixth network cluster is uniquely produced by NCD. In previous factor-analytic studies, the existence of a 6^th^ factor has been posited based on factor decompositions of the NEO-PI-R in large international samples including the Dutch population [27]. Also, a 6-FS has been identified in samples in which a modified version of the NEO-PI-R (Honesty/Humility-Emotionality-Agreeableness-Conscientiousness-Openness (HEXACO) model of Ashton and Lee [28]) that contains additional facets or items referring to normative (e.g. moral) judgments with respect to self and others. This sixth factor has been referred to as “Honesty/Humility”. Previous studies have shown that items and facets that are part of this sixth factor correspond to those of the Agreeableness factor of the Five Factor Model [29]. The items we found in the sixth network cluster are fewer than those described for Honesty-Humility. A difference in content is difficult to establish since HEXACO items differ from the NEO-PI-R items. Considering the nature of its items, however, it is possible that the sixth network cluster represents an Honesty/Humility cluster. Alternatively, the sixth cluster may just represent and ‘error-term’ of which the content is optimized to produce an optimal match of the remaining clusters with the five factors of the NEO-PI-R. In future studies, the existence of a sixth (Honesty) network cluster can be more specifically examined by matching HEXACO network clusters to HEXACO dimensions.

### The Personality Web as a developmental structure

The idea that personality develops towards maturity along certain paths or sequences of personality traits that are attained in the course of life has extensive support from studies of healthy personality (e.g. [30]–[32]). Research has shown that high levels of neuroticism put constraints to the maximum levels of agreeableness that can be reached [33]. A failure of higher-order (character) dimensions to develop has been linked directly to the presence of personality disorders [34]. Specific profiles of personality factor scores can be given for all personality disorders of the DSM-IV-TR [34]–[36]. These disorders all involve a failure of higher-order functions to develop [34]. A hierarchical structure of personality is further backed by findings from neuroimaging research showing that patients with personality disorders show deficits in higher-order brain areas such as the prefrontal cortex [37], [38]. So far, however, an integrative and empirical view of personality development in terms of developmental paths or sequences of traits has been lacking from the international literature. The six-cluster “Personality Web” that we derived at item- and cluster levels may qualify as such a view (Figures 5, 6). It describes possible routes that subjects may take during personality development. In such Webs, singular items and clusters can be identified that may play a crucial role in mediating the growth of healthy personalities. For instance, highly connected items (hubs) are crucial in keeping the Personality Web together and facilitating the communication between the various personality traits during development. Additionally, items with high betweenness centrality (i.e. that lie in between two large clusters) are central in mediating the communication between two clusters, as may occur during personality development. In general, singular network elements represent higher-order integrative structures that make complex networks vulnerable to damage [39]. If important nodes in the Personality Web fail to develop properly (e.g. if the neural correlates of hub traits are selectively eliminated as a result of a seizure or certain unfavorable environmental factors), this may cause a closing down of certain developmental routes, resulting in a massive dysfunctioning of human personality. In our dataset, items with the highest degrees (hubs) were mainly located within the extraversion and to a lesser degree the neuroticism cluster (Table S2). Since these hubs are all interconnected within the same cluster, these clusters form so-called ‘rich clubs’ of highly connected items [40]. In neuroscience, rich clubs represent higher-level integrative structures that serve to generate global representations of the environment [41]. The rich-club items of the NEO-PI-R connected with items from all other personality clusters. Hence, the extraversion cluster and neuroticism clusters can be considered singular clusters with respect to integrating information from all other personality clusters. Since extraversion and neuroticism are crucially involved in the regulation of positive and negative affect, respectively [42], [43], the possibility exists that neuroticism and extraversion dimensions represent higher-order integrative structures such as rich clubs, which are required for healthy affect regulation. At cluster level (Figure 6), the extraversion and neuroticism clusters did not stand out as major hubs, but the weights of the links surrounding the neuroticism and extraversion clusters were relatively strong, which can be due to the strong connectivity of the constituent items of these clusters. The extraversion, 6^th^ cluster and conscientiousness clusters showed highest betweenness centralities, i.e. were disproportionately involved in mediating traffic between other clusters. Hence, these three clusters seem to form an intermediate level within the personality structure that mediates the communication between the neuroticism cluster on the one hand and the agreeableness and openness to experience analogs on the other. The neuroticism cluster was the only node in the network that produced inhibitory effects (Figure 6). Since personality disorders according to the DSM-IV-TR are characterized by high levels of neuroticism and low levels of agreeableness and openness [35], [36], this makes for the hypothesis that agreeableness and openness scores are constrained by neuroticism indirectly through a direct effect on an intermediate developmental level constituting the clusters of extraversion, conscientiousness and perhaps the 6^th^ cluster. Since extraversion and neuroticism are central in affect regulation, the attained level of agreeableness and openness may largely be the net result of a balance between positive and negative affect regulation within the context of conscientiousness (Figure 6). In summary, the singular nature of the extraversion and neuroticism rich clubs makes it likely that a dysbalance in the development of these clusters produces an important impairment of normal personality development. Future studies in patient with personality disorders should learn the value of this hypothesis.

### Limitations and future directions

We have shown that the network (community) structure of data derived from multidimensional questionnaires can be optimized with respect to the FS of such datasets. However, factor analysis is prone on its own inaccuracies and mistakes. Thus, the network cluster decomposition may be biased by factor analytic results. Factor analysis is generally considered to be an “objective” technique, which examines observed covariance in datasets. The cut-off points used for the significance of factor loadings and the inspection of a screeplot may, however, be considered rather arbitrary [5]. Hence, other methods should be applied to independently examine the cluster structure of networks. Such techniques may involve different cluster algorithms, or other methods of determining the likelihood of network links, e.g. by using weight filtering techniques [44]. However, the use of weight filters is limited in correlational networks, since the weight distribution in these networks is approximately normal and weight filtering works best using non-normal distributions. Hence, the current approach of using the factor structure of correlational datasets as a template onto which the network community structure is optimized is among the most data-driven techniques that is currently available for network structure optimization.

The current study employed the Wakita-Tsurumi NCD algorithm because this technique has a strong theoretical match with principal component analysis and works for networks with large as well as relatively small numbers of nodes [23]. The Wakita-Tsurumi algorithm that we used is a non-weighted, non-signed, non-hierarchical clustering algorithm. This means that the weights of links (correlation strengths) and the signs of the correlation coefficients (positive or negative) are not used to define the modularity of networks. Additionally, network hierarchies are not detected. It is possible that different cluster algorithms that take these measures into account can provide additional information, and perhaps show equal or better matches with FSs (e.g. [26]). Specifically, clustering algorithms that examine hierarchical relationships seem to be promising instruments for future studies examining the scalable structure of Personality Webs, identifying facets and defining key areas of vulnerability of the Web (see above). Such techniques have proven their use in biological data, where they have shown interesting results for patterns of gene co-expression and brain activation [26].

Some remarks need to be made with respect to studies of the network structure of phenotypical data (questionnaires). In brain data or genetics, it is important to distinguish between nodes (genes, neurons or voxels) that show all positive (excitatory) or all negative (inhibitory) interrelations. In phenotypical data, however, such divisions are not straightforward. For instance, one item may ask whether a subjects likes bungee-jumping, whereas another item may ask whether a person dislikes taking risks. These items will have item scores that are likely to be negatively correlated, although they both attempt to measure the same underlying global trait (e.g. openness to experience). If such items would be clustered into different clusters (e.g. using signed cluster analyses), that would add little to the knowledge of the cluster structure of personality and more likely reveal peculiarities in the way the various questions are phrased. Hence, the sign of the correlations is of lesser importance in phenotypical studies than in biological studies. A similar language problem may bias the detection of hierarchies, which may turn out to represent either more general or more specific phrasings while testing for the same basic trait.

In summary, some level of caution is advised when interpreting the results of network cluster algorithms at the phenomenological level. It is important to attribute a correct amount of value to the information given by the singularity of certain nodes of the Personality Web. However, the NEO-PI-R is a very thoroughly studied questionnaire in which redundant questions that explain little additional variance in factor scores have been removed. Hence, it seems acceptable to regard hub-items in the NEO-PI-R as genuine high-degree connectors, and not as the product of badly phrased questions that correlate with many other item scores. The presence of a small-world structure in the NEO-PI-R network seems to point in the direction of a biologically plausible network [45], [46]. To further increase the validity of our findings, we included a fairly large number of subjects, especially when compared to other non-standard samples. Finally, we explicitly chose a non-weighted algorithm like Wakita-Tsurumi for the current study, since this allowed us to systematically vary the weights of network links during the incremental pruning technique. This enabled a detailed study of the effects of different connectivity strengths on the cluster structure of the NEO network and to determine its optimal network structure. No prior assumptions were made with respect to the existence of small-world structures or hierarchies so as not to overestimate the information content of our data. Despite such measures, however, biologically plausible structures did emerge from the data. It would therefore be interesting to compare the phenotypical network structure of personality to the structural and functional connectomes of the human brain.

### Conclusion

Network analysis of phenotypical personality data can be used to construct a Personality Web. Such webs are powerful tools for studies of normal personality development and personality disorders. A translation can be made from previous factor analytic findings to network-based descriptions of human personality. This is an exciting new avenue that has the potential to change our view of both healthy human functioning and disease. Network science provides a solid theoretical framework for studies of human personality. Since the human brain has a clear multimodular hierarchic network structure [47], it can be expected that the phenotypical correlates of the human brain (such a personality scores) have a comparable network structure of their own. Chances of finding meaningful relationships between neurophysiological and phenotypical levels might improve if one would study such relationships using network theory as a uniform, and perhaps unifying method.

## Supporting Information

Table S1 **Item level factor structures, with same specifications as in** **Table 1** **except that factor loadings >0.13 are shown.**
(XLSX)

Table S2 **Cluster contents and node-specific network metrics for the item-level network graph described in** **Figure 5** **.**
(XLSX)
