# Correction: The Network Structure of Human Personality According to the NEO-PI-R: Matching Network Community Structure to Factor Structure

**DOI:** 10.1371/annotation/519bd131-1b7e-4cf9-8d86-bcd923cb9435

**Published:** 2013-08-09

**Authors:** Rutger Goekoop, Jaap G. Goekoop, H. Steven Scholte

The current funding statement is inaccurate. The correct funding statement is the following:

Research was performed at PsyQ Psychomedical Programs in the Hague, an institution dedicated to providing specialized mental care. PsyQ allows its clinicians to reserve some of their time for research instead of health care. Data were obtained from healthy subjects without charge. The funders had no role in study design, data collection and analysis, decision to publish, or preparation of the manuscript. 

